# Translation and cognitive testing of the Italian Integrated Palliative Outcome Scale (IPOS) among patients and healthcare professionals

**DOI:** 10.1371/journal.pone.0208536

**Published:** 2019-01-02

**Authors:** Simone Veronese, Elisa Rabitti, Massimo Costantini, Alessandro Valle, Irene Higginson

**Affiliations:** 1 Fondazione FARO onlus, Torino, Italy; 2 Psycho-Oncology Unit, Azienda USL di Reggio Emilia–IRCCS, Reggio Emilia, Italy; 3 Scientific Directorate, Azienda USL di Reggio Emilia–IRCCS, Reggio Emilia, Italy; 4 Cicely Saunders Institute for Palliative Care, Policy and Rehabilitation, King’s College London, London, United Kingdom; University of Auckland, NEW ZEALAND

## Abstract

**Background:**

Outcome measurement is fundamental to assess needs and priority of care in palliative care settings. The Integrated Palliative care Outcome Scale (IPOS) was developed from earlier versions of this tool. Its use is encouraged to ameliorate the assessment of individual outcomes in palliative care settings. This study aimed to translate and culturally adapt IPOS into Italian, and explore its face and content validity.

**Methods:**

After forward-backward translation, a qualitative study explored the views of and cognitive processes used by respondents. We conducted individual semi structured interviews with 21 patients admitted to two palliative care services, from hospitals, hospices and the community, and focus groups with 12 professionals working in multidisciplinary palliative care teams and used thematic analysis. The results were integrated in a final audit, including the project team and the original POS developers, to refine the final format of the tool.

**Results:**

We conducted 21 face to face cognitive interviews with patients, and 2 focus groups with 14 professionals. Patients and professionals felt content and format of IPOS appropriate and feasible, and not burdensome. Some layout problems were raised leading to adaptation. Main issues regarded: clarifying the meaning of choices and some cultural interpretation of some questions and response options and interpretation of some instructions. We proposed using some new terms as more appropriate and comprehensive in our context, such as replacing the term “family” with “dear ones”. The items that appeared unchanged from the previously validated Italian POS were left unmodified to maintain coherence.

**Conclusions:**

The Italian IPOS, in its four versions directed to patients or staff and with a recall period of 3 or 7 days, has face and content validity for use in clinical settings and is ready for further psychometric and clinimetric validation.

## Background

The core of the WHO definition of palliative care [1] is to *prevent*, *assess and impeccably treat symptoms*, *and psychosocial and spiritual suffering*. To achieve this it is imperative to investigate patients’ outcomes. Many outcome measures explore some of these issues, but most are general and not specifically directed to the circumstances of palliative care patients, although some are more specific. Among them the POS family of patient reported outcomes (PROMs) is widely used, valid, reliable and continuously developed [2].

PROMs need to be translated and validated in the language of use in order to maintain the specific properties and to reliably report the authentic meaning of the explored domains [3]Recently an Italian version of POS core questionnaire has been validated [4] This version is now fully available for clinical and research settings. Due to the nature of the tool, that is modular, meaning that it is composed by a POS core, exploring pain, other symptoms and psychosocial, existential and information needs, it needs to be integrated by specific optional symptom lists that were developed for a number of patients’ groups (www.POS-PAL.org).

In order to overcome the difficulties to combine the modules a new integrated form of POS core, that includes a more comprehensive symptom list, was produced, named IPOS (Integrated Palliative Outcome Scale). This form differs from the original POS for other details: it starts with open questions, asking the patients what were the main problems or concerns they had to face in the chosen period of time. The list of symptoms (the most prevalent in palliative care) ends with three empty spaces inviting participants to add and rate any other symptoms.

IPOS has been validated for clinical use in English and German [5]. In this study cognitive individual interviews with patients cared for in the UK and in Germany were performed. Overall acceptance of IPOS was defined as good. Some issues were raised by participants about wording, length of answers and judgement difficulties, that were managed by the authors through discussion on cultural perspectives and item’s meaning. A refined version is now available in both languages. A full validation of psychometric properties is now ongoing in both United Kingdom and Germany.

This study aimed to translate IPOS in Italian, ensure appropriate cultural adaptation and determine and improve its face and content validity using cognitive testing. We considered both the patient and staff versions of IPOS.

## Methods

### Design

We followed the guidelines reported in the Manual for cross-cultural adaptation and psychometric validation of the POS development group, [6]. These were built from other well established standard translation and validation guidelines, such as those from the European Organisation for Research and Treatment of Cancer-EORTC [3].

**Fig 1:**
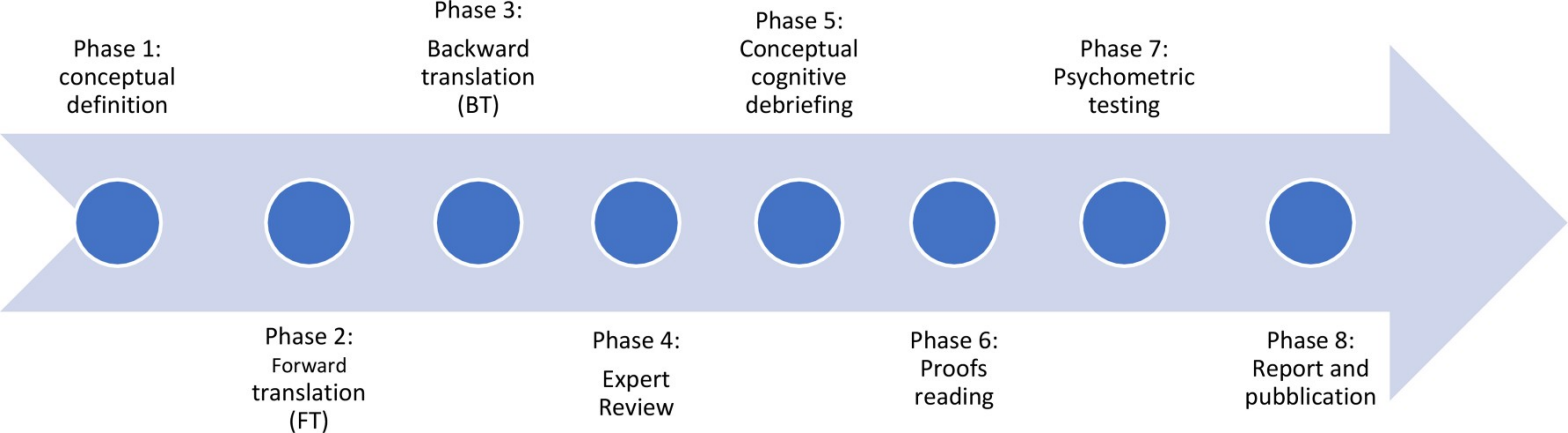
Flow chart from The Palliative care Outcome Scale (POS) Manual for cross-cultural adaptation and psychometric validation source.

The POS Manual consists of a eight phased process: Conceptual definition (1), Forward (2) and Backward (3) translation, Expert review (4), Conceptual cognitive debriefing (5), Proof reading—validation process audit (6), psychometric testing (7) and report and publication (8) (see Fig 1 for the flow chart of phases).

In this study, given that POS in Italian already existed, we focussed on phases 2-3-4-5 and 6:

### Translation

The original English IPOS versions were translated in Italian using the forward-backward translation process (Phase 2 and 3). The forward translation was performed by two independent Italian researchers, expert in palliative care. A first output, built after a comparison and agreement between the translators, called IPOS_ITA_FORWARD, was sent to an English mother tongue, Italian fluent speaking, translator who, in blind, produced a new English tool labelled IPOS_ITA_BACKWARD. The latter was forwarded to the original IPOS English authors for comments and remarks. Minor changes were raised and discussed between the English and Italian team of researchers (phase 4).

After this expert review phase, the Italian authors applied the required changes, creating an edited adaptation of the tool: the IPOS_ITA FOR COGNITIVE patient and staff versions. These new forms were used in the present study, that represents the conceptual cognitive debriefing phase of the Italian validation (phase 5).

### Cognitive testing

Cognitive testing sometimes referred to as cognitive or interview debriefing, is a term to describe a qualitative pretesting of the new IPOS in the target language, ensuring that the original instructions, items and scoring materials are clearly expressed. Two interviewers are needed.

This field test of the new tool uses the pre-final version with patients and staff. These groups are independent and therefore happen at different times. Each subject first completes the questionnaire, and is then asked about their thoughts on what was meant by each item and their response. Both the meaning of the items and responses are to be explored. This retrospective approach provides useful data regarding how an individual person interprets the items on the questionnaire, as well as their overall comprehension of the measure. It does not, however, addresses the construct validity, reliability or item response patterns. These will be studied in phase VII. The described process provides for assessment of quality in the content validity [6].

#### Setting and participants

Two North Italian specialist palliative care units were involved in this study. One unit has a clinical palliative care ward in an acute oncology hospital (IRCCS Reggio Emilia). The second has a palliative home care service and two hospice wards (FARO Foundation, Torino). Both teams are multiprofessional, include cancer and non-cancer patients and are involved in clinical activities (inpatient units, outpatient consultations, home care services and primary care support) in research and education (Quality of life and PROMs development, palliative care for non cancer patients and symptoms control).

To obtain validation of the patients’ version, semi-structured individual interviews with patients receiving specialist palliative care were conducted. For the staff version one focus group in each of the two clinical centres were run. Participants were professionals working in the two multidisciplinary palliative care teams (MPCT).

For the individual interviews to patients a purposive sampling technique was chosen, with the aim to select participants from different clinical settings (home care, hospice or hospital wards) and geographical settings, with different primary diagnosis who could provide in-depth insight about the tool structure. For this reason, we excluded patients whose clinicians deemed to be too ill or distressed.

For the focus groups we included professionals who work daily in the field of palliative care, with experience in the use of PROMs such as IPOS or POS.

Inclusion criteria for the patient interviews were: age > 18, Italian as first language, no relevant cognitive impairment and capacity to give consent. For the focus groups participants had to be professionals expert in palliative care (at least 3 years of clinical practise in the field), fluent Italian speaking and actually working in one of the two MPCT. Exclusion criteria for the patients were being too debilitated or cognitively impaired to sustain an interview or not willing to participate.

Patients were selected through a first identification from the clinical nurse who was in charge of their care and knew the inclusion-exclusion criteria, followed by a discussion with the research team. Researcher then got in touch with the potential participants describing the study and asking their consent to participate.

Professionals were selected by the research team according to their previous experience and interest on the use of IPOS and PROMs, and then asked to provide consent and to participate to the study.

Interviews were conducted where the patients were receiving care, in one of the 3 described settings. Focus groups (FG) were run in the two working settings.

The interviewers and focus groups conductors were expert researchers working in the MPCT previously trained according to the “Topic Guide for Integrated Palliative Care Outcome Scale (IPOS) Pilot survey (Topic Guide)” provided by the IPOS translation team. This tool prompts towards the general aspects of the test, comprehension, retrieval, judgement and response process used by the interviewees.

The trained interviewers were one nurse working in the palliative care ward and 3 psychologists working in the hospice wards and home care team. All of them knew the interviewed patients and participated to their preselection.

The two PI supervised the data collection, provided education training to the interviewers and took part to some interviews and focus groups.

#### Study procedures

**Fig 2:**
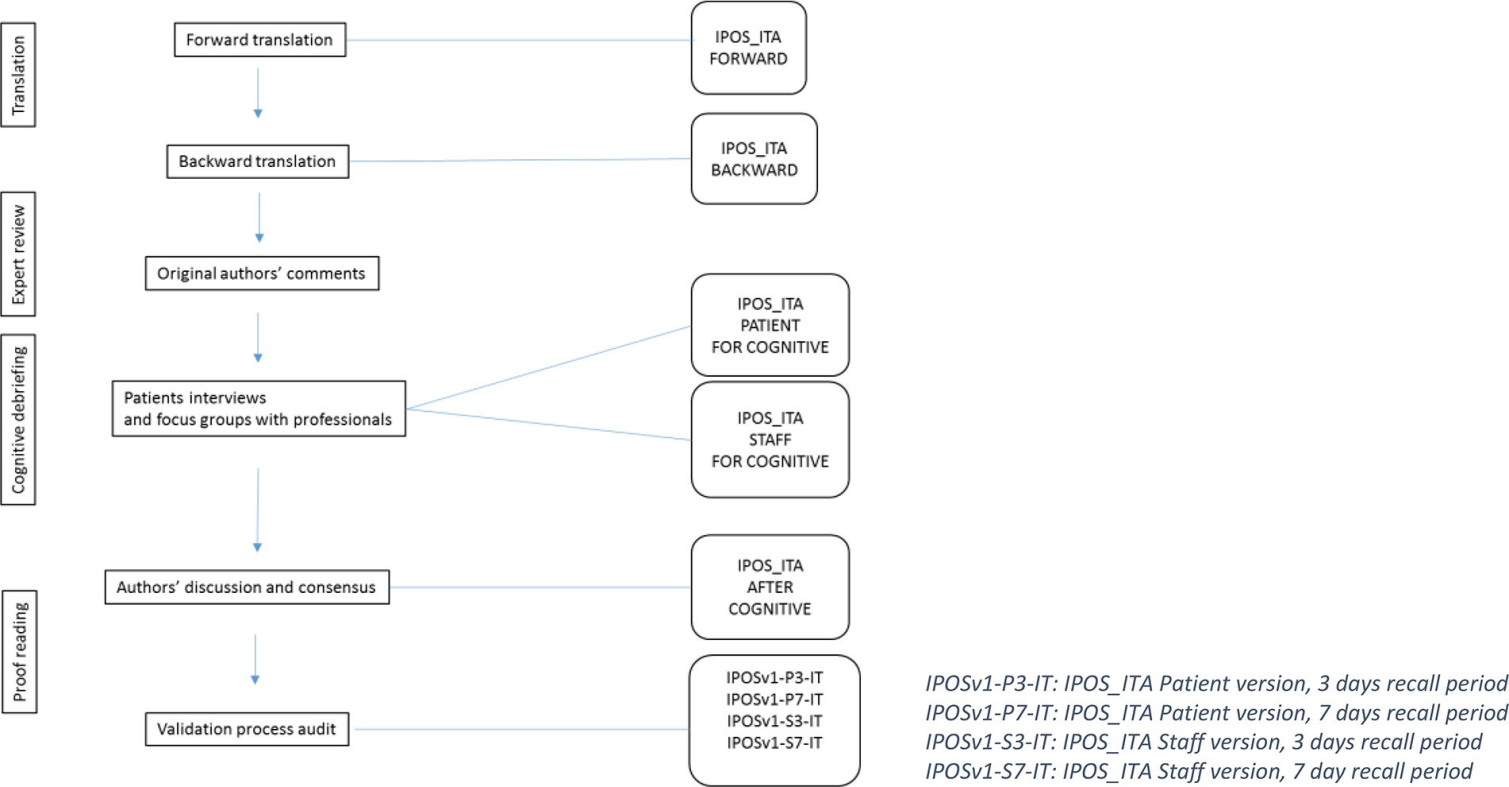
Stages of the translation and cognitive validation process of IPOS_ITA.

The overall study design is shown in the flow chart exposed in Fig 2. For the purpose of this study the 7 days versions of IPOS were adopted. After the translation process the cognitive phase was performed, representing the phase 5 of “The Palliative care Outcome Scale (POS) Manual for cross-cultural adaptation and psychometric validation”. (S2 Appendix)

In the individual interviews participants filled the IPOS and were hinted to read and answer the questions one at a time, then stop and talk about each question before moving onto the next. Interviewees were invited to ‘think out loud’, explaining how they arrived at the answers, rather than the result of the answer itself. They were also asked to report any thoughts or views they might have had about the questions. According to the Topic guide manual, conductors explored general views on the tool as well as comprehension, retrieval, judgement and other potential factors impacting the cognitive evaluation.

All the selected patients invited to the study consented to participate. The interviews took on average 45 minutes (range 30–60).

It was not possible to calculate the time of IPOS_ITA completion since the interview focused on each single item, with a thorough discussion of the comprehension and acceptability of the wording. All the interviews were concluded without evidence of distress of the participants, none of them asked to interrupt for excessive burden and no questions were declined or deemed invasive.

The two focus groups involved 12 professionals with expertise in palliative care. The events were run separately and conducted by the principal investigators of the two study centers. All participants had previous experience of IPOS_ITA use, some conducted interviews with patients, others used it in clinical practise. Both focus groups lasted about 1 hour.

In the focus groups participants were asked to go through the IPOS staff version, with the same approach suggested to the patients. They were also invited to provide reflections on how they thought their patients might have issues in understanding and interpreting the tool’s questions and answers.

Data from the events were registered by written field notes, or audio registrations, based on the researcher’s choice. In all cases comments were recorded. The transcriptions were not translated in English, only the emerged issues were reported in English to the original authors for the following validation audit.

#### Data analysis

Data analysis was individually performed by the two PI of the study, through reading and re-reading of the transcriptions, focusing on each item. All the IPOS_ITA fields, questions and answers, were checked in terms of wording, acceptability, ambiguous meaning, as well as format and layout issues. Using the Topic Guide as a reference framework, a thematic analysis [7] allowed us to focus on the most relevant issues.

The thematic analysis was performed using an informal approach based on questions and answers. Any difficulties emerged during the filling of IPOS or in the discussion with participants were considered as a possible issue and coded to be reviewed. The categories (see S1 Appendix) used to perform the analysis were: comprehension (what does the respondent believe the question to be asking), retrieval (Could they recall the information required by the question? Was the time frame suitable?), judgement (Is the respondent able to make an evaluation based on the information recalled?), response (Is the respondent able to map their internally generated answer to a response option?) and others, (including additional comments, the questionnaire overall impression, questions to be removed, layout issues).

The emerged themes were compared and aggregated for each question and each category in both centres, through consensus between the PI. The aim was to highlight the problems or perplexities emerging from the tool, in order to refine and edit it in a new output (IPOS_ITA_AFTER COGNITIVE) to be sent to the English author for proof reading, comments and remarks.

A final validation audit, involving the PI, the main English author (IH) and the author of POS Italian versions (MC), was set up to merge all the issues and arrange the final IPOS_ITA tool to be published on the official website.

The audit process aimed to check any single change, throughout the match and comparison among all the items of the other following POS family versions: original English IPOS, Italian POS and the new and final IPOS_ITA versions.

#### Ethics

The study protocol was approved by the Ethical Committees of the two research centres (CE Interaziendale A.O.U. Città della Salute e della Scienza di Torino, reference number CS\599 protocol 0070308, and CE Provinciale Arcispedale S. Maria Nuova di Reggio Emilia, protocol 2015/0021569).

Confidentiality of the data is guaranteed by anonymity of the participants through a coding process. Sensible data are securely stored by the PI. To preserve patients and professionals by possible psychological distress they could stop the interviews at any time or decline invasive questions.

Written consent to the study participation was obtained by all participants.

## Results

### Demographic and clinical features

From July 2015 and February 2016, 21 individual interviews with patients (11 FARO, 10 IRCCS) and 2 focus groups (one in FARO 7 participants, one in IRCCS with 5 participants) were performed. Interviewees’ characteristics are reported in Table 1, FG participants’ in Table 2.

**Table 1:** Patients' demographics and clinical data.

Types of participants	Recruitment setting	Patients interviewed	Age	Gender
Patients with solid cancer	Hospice	3	Range: 62–77 M = 69,7 SD = 7,5	3 Females
	Home care	2	Range: 63–75 M = 69 SD = 8,5	1 Male 1 Female
	Hospital	10	Range: 43–74 M = 57,3 SD = 11,65	5 Males 5 Females
Patiens with hematological cancer	Hospice	3	Range: 66–81 M = 73,7 SD = 7,5	2 Males 1 Female
Patients with COPD	Home care	1	83	1 Male
Patients with IPF	Home care	2	Range: 72–76 M = 74 SD = 2,8	2 Males

M = Mean; SD = Standard Deviation

**Table 2:** Focus Groups participants’ role and background.

Profession	Place of work	Role in the study
Nurse	oncological ward	interviewer
Physician	oncological ward	co-interviewer
Physician	oncological ward	co-interviewer
Physician	oncological ward	clinician
Psychologist	IRCC centre	clinician
Psychologist	hospice	interviewer
Nurse	hospice	clinician
Nurse	home care	clinician
Nurse	hospice	clinician
Psychologist	hospice	interviewer
Psychologist	hospice & home care	interviewer
Psychologist	home care	interviewer
Physician	hospice & home care	conductor of the Focus Group & interviewer
Psychologist	oncological ward & home care	conductor of the Focus Group & co-interviewer

### Findings from the cognitive debriefing phase and adaptation of IPOS_ITA version

Overall this study found that all the interviews were concluded without difficulties or fatigue for the patients. No interviews had to be interrupted for patients’ distress or for lack of comprehension or clearness of the items. Professionals reported a sense of “feeling at ease” in the use of the tool, and an overall satisfaction about its functionality and usefulness.

However, a number of key points and critical issues regarding the Italian wording, meaning, interpretation and layout, emerged. These and subsequent changes are detailed in appendix. (S3 Appendix)

#### Layout and presentation

In the interviews and focus groups some difficulties in reading and filling the forms were complained about by participants, therefore some layout changes were made to make the tool more easy to complete, for example increasing the types font and reprinting with wider interline space.

Between Q5 and Q6 the order of the possible answers reverses because of the nature of the questions (Q5, asks about depression, a negative symptom, Q6 is about being at peace, a positive feeling of wellbeing). Thus, Q3-Q5 are rated Not at all (0) → to Always (4), Q6-Q8 is the opposite, Always (0) → Not at all (4). This caused difficulties since some patients did not notice the change reporting a wrong answer.

*“The answers are not well done because I get confused with the following question”* a female (aged 50) patient from the hospital ward.

*“I want to underline the risk of wrong answers due to the positioning in the same column of answers with opposite meaning”* (Professional working in the home palliative care team)

A change in the output was made in order to separate the different blocks of questions and reduce the possible errors.

#### Content, acceptability, comprehension and meaning

Many positive comments were made about the choice of prioritizing open questions rather than starting with a problem checklist.

*“I was positively surprised when you asked me about my problems before asking me if I were in pain”* (a patient in the hospice ward)

The 7 day version of the IPOS adopted in the study required that the patients had to rate symptoms intensity that could have had fluctuations and different impact on their experience over 7 days. This caused problems of retrieval and difficulties in choosing an answer.

*“I had shortness of breath every day in the last week*, *but pain for example varied*. *So I am not sure what to choose”* (a patient with IPF, home care)

Professionals reported difficulties in estimating the degree which patients were affected by symptoms explored in Q2. They did not know if they had to report the highest intensity or report a mean value.

*“When they asked me about what to choose I was not sure what to say*. *In general in palliative care we tend to consider the highest degree of a problem*, *but here I just didn’t know*. *Maybe they had an awful symptom for few hours in a week*, *should they call it unbearable*? *Or because it went away quickly and didn’t come back it was rather mild or moderate*?*” (*Professional working in the home palliative care team)

For this reason further explanation in brackets was added, suggesting that in the case of fluctuating symptoms a mean value could be entered.

Questions about the symptoms were found very clear and well-constructed. The proposed answers did not create confusion and participants could easily reflect on their experiences. The only exception was the item on sore or dry mouth.

*“If I have a slight sore mouth and a severe dry mouth*, *which one I sign*?*” (*female patient in the hospice ward)

Being a double choice of two very common and unpleasant symptoms, some patients debated if they had to choose between the 2 symptoms or rate the worst, or deny both since one was present but the other not.

One patient suggested dividing the question in two:

*“Dry mouth and pain in the mouth are 2 completely different things and should be divided”* (Patient in the hospital ward)

In order to ask a single question comprehensive of both symptoms, a new formulation was proposed, according to the NEURO_IPOS symptom checklist recently published [8].

Other changes were applied to the possible answers in Q3-Q8 questions: the word “occasionally–occasionalmente” was changed in “rarely- raramente”, since in Italian this makes more sense. This is consistent with the Italian version of POS [4]. Furthermore in Italian it is hard to difference between “occasionally” and “sometimes”, whereas “rarely” is more distinguishable.

*“I was undecided and doubtful between occasionally and sometimes since I did not recall the exact difference”* patient, home care)

Some difficulties emerged in the second part of the tool, regarding the psychosocial, existential and social issues.

Question 3 asked about “feeling anxious or worried about the illness or treatment”. This generated hesitation due to the multiple choices exploring anxiety, worries and being directed towards the illness and the treatments. Several patients and professionals reflected on these issues from different perspectives. Patients were more concerned about the difference between being worried about the illness or the treatments: some said they were worried about the disease and not the therapies or vice versa and did not know how to answer. Professionals perceived the word anxiety as a diagnostic category, see for example DSM-5 manual [9], that might cause misunderstanding.

“I fear that the term Anxiety refers to a pathological condition, I wouldn’t like that they felt more ill than they already are. Being worried can be more acceptable for our frail patients” (Psychologist working in hospice)

In a focus group a new formulation was proposed: “have you been worried for your health?” that was felt to be more clear and acceptable and including both options. During the validation process audit this rewording was rejected, because the original IPOS included all the different choices, explored in multiple needs assessment of palliative care patients and to maintain congruence with the fully validated POS_ITA version.

Question 4 explores how much family or friends have been anxious or worried for the patient. According to the previous considerations about difficulties related to multiple choices, a proposal of change was “Have your dear ones been worried for you?”

“I think that we should either split the question for family and friends or keep it together focusing on the people important for the patient” (participant of a Focus Group)

“What if my family are ok but my friends are extremely suffering for my condition? My girlfriend seems well adapted to my condition, but my friends and one of my son who lives in South America are very concerned” (Patient, hospital ward)

For the same thoughts of Q3 the double answer “anxious or worried” was maintained, but a new simplified and inclusive wording was adopted to substitute “family and friends”. It seems that “dear ones” in the Italian setting is more clear, inclusive and acceptable.

Question 5, exploring low mood, was maintained unchanged using “have you been feeling depressed?” even though some issues, as for anxiety, were raised. Depression can be seen as a stigma in Italian culture as well as in Germany [5],professionals tend to see it as a diagnostic category sometimes covering a physiological decrease of the mood state. However it was highlighted how this question was shown useful in the screening for potentially clinical significant depression that could lead to specific therapeutic approach [10].

Question 6 explores the existential-spiritual domain of being at peace. Some patient reported the question as too generic or non-specific:

*“I would reformulate the question avoiding the word “at peace” that is unusual*. *I’d rather use–serene- or -feel well-“(P*atient, hospice ward)

*“What does it mean being at peace*? *Only the dead are at peace”*. (patient, home care).

*“Some patients think this concept of peace is for religious people*, *for other peace is just the opposite of war*, *above all those elderly who lived the war*.*”* (Professional in a focus group).

For others this term was “too much religious”. Participants suggested “serene” which is very used in Italian. It is not “being calm” which is seen as the opposite of “worried”, therefore explored in Q3. A new version “have you been at peace within yourself” was adopted since this is coherent with POS_ITA.

Question 7 explores how much patients were able to share their feelings with family and friends. According to the variation proposed in Q4 “your loved ones” was proposed. This new formulation encountered the authors’ favourable opinion because it is inclusive of any person loved by the patient independently from the boundaries or formal labels.

No particular issues came out for the information item except for a minor word change exposed in the table of the results (S3 Appendix). The last question exploring social, practical, financial and personal problems caused some misunderstanding:

*“The question is vague*. *What kind of problems are these*? *Of health care*? *I would distinguish personal and financial”*. (Patient, hospital ward). In Italian the words “financial or personal” were felt confusing for both participants and professionals. Economical seems more clear in our cultural setting than the word financial, which is linked to stock exchange meaning. Social seems to explore directly the need, personal seems not to reach the point. Many patients asked what was meant with personal, probably thinking about personality-behaviour concerns.

According to the these findings, new IPOS_ITA versions (IPOS_ITA after cognitive) were made and sent to the original authors for revision and approval.

To obtain a final revised version that include the findings from the cognitive phase of IPOS_ITA, maintain the IPOS_English structure as much as possible and integrate the POS_ITA items whenever possible, a proof reading audit (phase 6 of the Manual, S1 appendix) among the researchers and the original authors was established. Some suggested changes proposed after the cognitive phase were accepted and maintained. Other issues could not be applied and therefore a common agreement was achieved. At the end of this process a final IPOS_ITA version was produced, including two patient versions and two staff versions (3 and 7 days of recall).

## Discussion

We were able to satisfactorily translate the IPOS, check its cultural adaptation and establish face and content validity. In cognitive interviews all participants found the Italian IPOS acceptable, feasible and not burdensome. None of them asked to interrupt the interview due to tiredness or difficulties due to their conditions. Professionals involved in the focus groups were motivated and reported positive feedback on the utility of the tool in the clinical setting. These findings are consistent with previously published similar work [5].

Testing the tool in 2 different palliative care Italian centres allowed the possibility to raise issues identifiable in different models of care, or specific for centre, as suggested by other authors [11].We recruited patients in different settings: the home care service, hospice ward and palliative care ward in acute hospital, allowing collecting point of view of participants influenced by their place of care. Our findings showed no major differences among these contexts, suggesting that IPOS explores domains shared across centres and settings. Some layout or data field problems, were easily solved by refining the graphical visual of the test. Another novel result from this study is that the cognitive exploration involved both patients and professional carers. This process enabled participants of the validation audit to blend the findings and issues into a comprehensive final version including the different point of views, which we believe increases the value of IPOS_ITA in clinical practice.

The sample of patients included those with cancer and non-cancer diagnoses. This reflects the trend of offering palliative care independently from disease group [12], but based on the unmet needs. No differences arouse from the two groups, confirming that IPOS_ITA is usable in palliative care independently by the primary diagnosis [13].

A key modification concerned how much the symptoms affected the patients over time (Q2). The proposed wording, and sub sequential scoring, did not allow participants to report clearly any fluctuations of the symptoms. This issue was raised in the German-English cognitive validation of IPOS, where patients had judgement problems reflecting replies, regarding the timeframe period over which symptoms were sought [5]. As a result, they produced a new IPOS version with an extended recall period of 7 days. Unfortunately, this solution did not meet our patients’ problems, since it was not just a matter of more days, but not what to choose when the symptom varied in intensity over time. To overcome this issue, we added a clarification sentence, that suggests indicating an average value in the case of fluctuating symptoms.

Our process was very effective in identifying and considering in detail the issues in the questionnaire and in exploring different solutions for individual issues. For example, we found that participants could feel confused when a question included double response options, but not always. The physical symptom list (Q2) third question asks about if and how the patient is affected by weakness or lack of energy. Here, both patients and professionals agreed that the question was clear, since the double choice strengthened the concept. However, question eight, regarding “oral problems” was found to be confusing; because the two proposed answers could be either present or not so. In the latter situation, it was difficult to choose one of the two options, and/or to rate the intensity.

In the audit, these issues were discussed and accepted: weakness and lack of energy remained (translated in Italian), but sore or dry mouth was changed in “Mouth problems”. This new formulation was proposed as possible change for both the Italian POS versions and the original English IPOS tool. This wording was then adopted in the new neurological version of IPOS recently developed and published [8].

In contrast, an opposite decision was made about Q3, which explores feeling anxious or worried about illness or treatment. In this formulation, there are four different possible choices. In our study, participants reported confusion and uncertainty about the possible answers. As a temporary result the authors proposed a simplified version of the question (Have you been worried for your health?) which seemed inclusive and straightforward. In the audit, after a thorough consultation, the original IPOS authors argued that the proposed version, although easier to understand, was missing components that patients judged important in the original tool development. A further consideration was in Q4, which explores the patient’s point of view about the concerns of family or friends around his or her condition. Participants in our study felt difficulties in choosing between family or friends, and sometimes even among familiars. We proposed changing to “dear ones” to substitute the double choice “family or friends”. The notion of “dear ones” (loved ones or close ones) seems comprehensive and understandable and could, we believe, be extended to the POS-TOOL family, and may be more inclusive of friends and supportive neighbours, as well as relationships such as gay, lesbian, bisexual.

Finally, our findings highlighted how difficult it can be to ask about depression (Q5) in some cultural contexts. Professionals, mainly those with a psychosocial background, were concerned to use a potential diagnostic label, which could be seen as pathological, whereas often severely ill patients may be just deeply sad. Some patients felt a sense of embarrassment related to this term. This is consistent with the findings of the German study where participants suggested rewording the question because the term “depressed” was not socially acceptable [5]. The problem may be related to a feeling of social stigma. Based on these thoughts the authors proposed to change the question asking if the patient felt sad or in low mood (to strengthen or clarify the meaning, not to create a double choice). During the audit the importance of screening for depression, using a clear and not ambiguous wording, was underlined, according to the published Clinical Decision Support Tool for the interpretation of and response to Palliative care Outcome Scale (POS) scores [10]. The final decision to maintain the word “depression” followed a balanced judgement between the reluctance of the participants to respond, or to submit such a question, and the evidence that a score higher than 2 can be a sensible cut off point, indicating a possible clinical depression worth to be further investigated and treated. Finally, the same formulation remains in the fully validated POS_ITA, confirming that the barriers are not insurmountable.

### Limitations

This study has several limitations. The qualitative nature of the research led to a small sample size, which was appropriate for the design [11], but, for its nature, not generalizable. Furthermore, our sample size was consistent with similar studies [5].

The focus groups were conducted by only 1 researcher due to resources limitations. This might have caused some data loss, even though accurate notes were taken by the conductors.

Although we were able to collect the view of patients and professionals, they sometimes differed in their views regarding the proposed amendments. We also wanted to maintain consistency with the original IPOS, to allow international comparison. Therefore, some modifications were a compromise between the different views. A next key step would be to perform a full assessment of the measurement properties of IPOS_ITA, including construct validity, reliability, and acceptability, including time to complete.

## Conclusions

This process of developing and considering the cultural adaptation of IPOS provided us with a rich understanding of how IPOS results may be used and interpreted in practice. We produced the IPOS tool in Italian language, maintaining the available four versions, for the patient and for the staff each with a 3 or 7 days recall time, as in the original English tool, and these are now freely available on the POS website (www.pos-pal.org). Our data suggest that IPOS_ITA has face and content validity for use in the clinical setting, although further formal psychometric validation is needed. Our findings of the interpretation of questions are useful for others translating and adopting IPOS into other languages and cultures, and also will be useful for the future development of IPOS, especially regarding double response questions and response options.

## Supporting information

S1 Appendix Topic guide for Integrated Palliative Care Outcome Scale (IPOS) Pilot survey (Phase I). (PDF)

S2 Appendix The Palliative care Outcome Scale (POS) Manual for cross-cultural adaptation and psychometric validation. (PDF)

S3 Appendix TABLE of RESULTS. (DOCX)

## Abbreviations

IPOS: Integrated Palliative care Outcome Scale
POS: Palliative care Outcome Scale
WHO: World Health Organization
PROMs: Patient Reported Outcome Measurements
EORTC: European Organisation for Research and Treatment of Cancer
PI: Principal Investigators of this study
MPCT: Multidisciplinary Palliative Care Teams
FG: Focus groups
COPD: Chronic Obstructive Pulmonary Disease
IPF: Idiopathic Pulmonary Fibrosis
