# Supplementary material for: Translation and cognitive testing of the Italian Integrated Palliative Outcome Scale (IPOS) among patients and healthcare professionals
Source: PLoS One. 2019 Jan 2;14(1):e0208536. doi: 10.1371/journal.pone.0208536 (PMC6314603; doi:10.1371/journal.pone.0208536)
Supplement: S1 Appendix — (PDF) [file pone.0208536.s001.pdf]

School of Medicine
at Guy's, King's
College and St
Thomas' Hospitals

Department of
Palliative Care, Policy &
Rehabilitation

Professor Irene Higginson
BMedSci BMBS FFPHM PhD FRCP
Head of Department
Professor Lynne Turner-Stokes
DM FRCP
Herbert Dunhill
Chair of Rehabilitation

Cicely Saunders Institute
Bessemer Road
Denmark Hill
London SE5 9PJ
Tel: +44 (0)20 7848 5516
Fax: +44 (0)20 7848 5517
www.kcl.ac.uk/palliative

KING'S College LONDON

Topic Guide

Integrated Palliative care Outcome Scale (IPOS)
Pilot survey (Phase I)

Objective:

- To explore the cognitive processes used by respondents when reading, interpreting and responding to items on the IPOS questionnaire

- *Introduction:*

  - Study purpose, confidentiality, able to stop at any time, decline questions

  - I'm going to show you a questionnaire and I want you to read & answer the questions one at a time
  - We will stop and talk about each question before moving onto the next
  - Please try to 'think out loud' as you read and answer the questions (*DEMONSTRATE*)
  - I will also ask you some more specific things about each question

  - Apologies of the questions get repetitive

  - In this study we are less interested in your answers to the questions, but *how you arrive* at the answers – what you think the question means, and the things you were thinking about when you chose your answer.

  - You can tell me *any* thoughts or views you might have about the questions

-- START RECORDING --

- *General:*

  - What were you thinking about when you answered that question?

  - I noticed you hesitated before giving your answer – what were you thinking about then?

- *Comprehension:* ***What does the respondent believe the question to be asking?***

  - What does the question mean to you, in your own words?

  - What does the word XXXXXX mean to you? (if certain words are thought to be problematic)

  - How easy or difficult was it to understand this question?

  - (If problem) How would you change this question?

- *Retrieval:* ***Could they recall the information required by the question? Was the time frame suitable?***

  - How well could you remember your experience when answering this question?

  - Was it easy or difficult to think about the past [week] when answering this question?

  - Would there be a different time period that would be easier to understand?

- *Judgement:* ***Is the respondent able to make an evaluation based on the information recalled?***

  - What were you thinking about when you answered this question?

  - How did you arrive at your answer to that question?

  - Was that easy or hard to arrive at your answer? Why do you say that?

  - How sure are you of the answer to this question?

- *Response:* ***Is the respondent able to map their internally generated answer to a response option?***

  - How did you choose your answer to this question?

  - Was it hard or easy to select an answer from the options given?

  - Did all options make sense for this question?

- *Other:*

  - Is there anything else you would like to say about this question? / The questionnaire as a whole?

  - Did you find any of the questions upsetting? / embarrassing? / inappropriate?

  - Are there any topics/questions that you would leave out of this questionnaire?

  - Are there any topics/questions that you would add to this questionnaire?

  - Do you have any thoughts about the way your answers were captured? (i.e. tablet/paper)

--- THANKS + STOP RECORDING ---

----------------------------------- COMPLETION OF DEMOGRAPHICS FORM -----------------------------------
