# Supplementary material for: Translation and cognitive testing of the Italian Integrated Palliative Outcome Scale (IPOS) among patients and healthcare professionals
Source: PLoS One. 2019 Jan 2;14(1):e0208536. doi: 10.1371/journal.pone.0208536 (PMC6314603; doi:10.1371/journal.pone.0208536)
Supplement: S2 Appendix — (PDF) [file pone.0208536.s002.pdf]

The Palliative care Outcome Scale (POS)
Manual for cross-cultural adaptation and psychometric validation

Author: Bárbara Antunes[1]

Contributors: Barbara Daveson[1], Christina Ramsenthaler[1], Hamid Benalia[1] and Pedro Lopes Ferreira[2].

Coordinators: Claudia Bausewein[1] and Irene J. Higginson[1]

On behalf of the POS Development Team
King's College London, Cicely Saunders Institute, Department of Palliative Care, Policy and Rehabilitation

Affiliations

[1] King's College London, Cicely Saunders Institute, Department of Palliative Care, Policy and Rehabilitation

[2] Centro de Estudos em Investigação e Saúde da Universidade de Coimbra

February 2013
Version two

Table of Contents

Foreword

Introduction

Phase I: Conceptual Definition

Phase II: Forward Translation (translation from the original english to target language)

Phase III: (Blind) Backward Translation (translation from the target language to english)

Phase IV: Expert Review

Phase V: Cognitive debriefing

Phase VI: Proof Reading

Phase VII: Psychometric testing

Phase VIII: Report and Publication

References

Acknowledgements

Appendices

Appendix A: Template summarizing General Information on participants
Appendix B: Template summarizing all phases of the process
Appendix C: Template for Final Reports (final step of each phase)
Appendix D: Template summarizing the synthesis of the two forward translations
Appendix E: Template summarizing the synthesis of the two backward translations
Appendix F: Psychometric (Pilot) testing report

You should cite this document as:

Antunes B, Daveson B, Ramsenthaler C, Benalia H, Ferreira PL, Bausewein C and Higginson IJ; The Palliative care Outcome Scale Manual for cross-cultural adaptation and psychometric validation, London, March 2012 (http://pos-pal.org/Resources.php)

Foreword

Since the first publication of Palliative care Outcome Scale (POS)[1] in 1999, interest in the adaptation and validation of POS has grown. At the time of this publication POS has been translated in more than 12 languages. This growth and interest has prompted the POS Development Team to produce this guidance for the cross-cultural adaptation and validation of the POS and all of its derivatives for use with adults. This guidance will help ensure the construction of excellent quality instruments, derived from universally accepted translation and validation standards regarding scale development.

On behalf of Professor Irene J Higginson, the creator of POS, and the POS Development Team, we hope you find this guidance useful and we welcome you to the global community of POS users working together to help advance outcome measurement and in particular the use of POS in palliative and end-of-life care.

Bárbara Antunes

This document is also available on line at:

pos-pal.org

Introduction

Cross-cultural adaptation and validation procedures create a version of the original scale in a target language that is conceptually equivalent to the source instrument and psychometrically valid to allow for data pooling and cross-national and cross-cultural comparisons. A standardised methodology[2-8] is required for this to be achieved, and the process is usually completed by a team of people with different skills. Careful planning is required in order to create a reliable measure for the target population. A rigorous adaptation and validation methodology ensures that the resulting measure describes the impact of a disease or its treatment in the context of different cultures in a similar manner.

> Box 1
> The process involves the adaptation of
> (1) instructions for the questionnaire,
> (2) all individual items,
> (3) response options and
> (4) the scoring documentation.
> Throughout this document when “the POS” or “the questionnaire” is referred to, it must be taken into consideration that we are referring to these four components.

This document outlines the complete methodology for cross-cultural adaptation and psychometric validation (see Appendix A) in relation to POS including POS version one, POS version two, the Palliative care Outcome Scale-Symptoms (POS-S) and the disease-specified versions: POS-S Renal, POS-S Parkinson, POS-S Multiple Sclerosis (Phases I - VII). Additional resources are also included to assist you in your adaptation and validation work (Please see Appendices). The POS Development Team based at King’s College London is a useful resource to aid you in your cross-cultural adaptation and psychometric validation work and has produced a number of quality assurance procedures and templates that can assist you.

> **Key points to remember when using this manual**
>
> ●This document was created specifically for the Palliative care Outcome Scale, a multidimensional scale, and the disease-specified versions
> ●The methodology described in this document is widely accepted, although not exhaustive of a translation and validation process. This document describes the required methodology to produce a good quality measure.
> ●Templates are provided in the Appendix to help maintain a good record of all the work in the different Phases
> ●Please take time to go through the references we provide for additional information

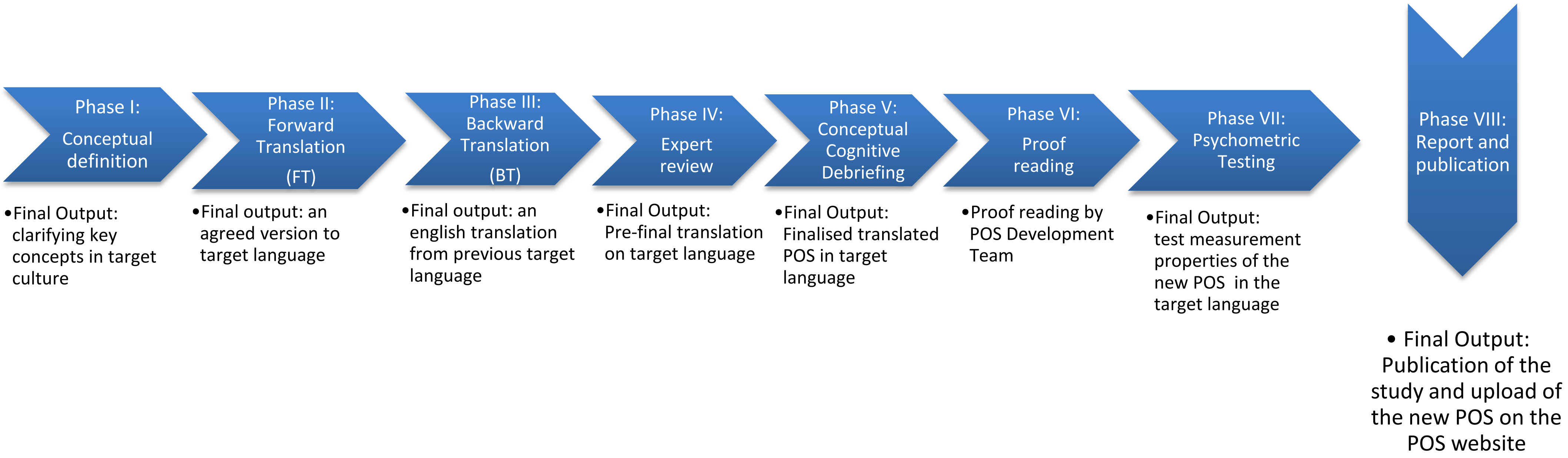

Phase I: Conceptual definition
•Final Output: clarifying key concepts in target culture
Phase II: Forward Translation (FT)
•Final output: an agreed version to target language
Phase III: Backward Translation (BT)
•Final output: an english translation from previous target language
Phase IV: Expert review
•Final Output: Pre-final translation on target language
Phase V: Conceptual Cognitive Debriefing
•Final Output: Finalised translated POS in target language
Phase VI: Proof reading
•Proof reading by POS Development Team
Phase VII: Psychometric Testing
•Final Output: test measurement properties of the new POS in the target language
Phase VIII: Report and publication
• Final Output: Publication of the study and upload of the new POS on the POS website

Phase I: Conceptual Definition or Equivalence

Achieving conceptual definition or equivalence involves appraising and clarifying the concepts investigated by each item of the original POS to ensure they will be equivalent in the target language (use Appendix B). This is important because the new measure will need to reflect palliative care concepts appropriate to the target culture. It may occur – and it does sometimes – that certain concepts are not recognised or are meaningless in a particular culture, so by defining the concepts of the original POS beforehand the team will be (1) better acquainted with the measure they are about to work with and (2) aware of some concepts that might need to be worked on in order to accommodate the values, beliefs and characteristics of the target population.

Three steps need to be completed to achieve conceptual definition/equivalence.

- Step one: brief review of the literature on the health-related quality of life issues in palliative care patients in the target culture. This will help the team to familiarise themselves with important concepts and issues specific to the target population.

- Step two: identify, analyse and define key concepts that underscore each item of the original POS by conducting semi-structured interviews with a purposive sample of palliative care professionals. It is important to go item by item.

- Step three: conduct an investigation of the concepts defined in Step two through two focus groups of the target population to ensure that those concepts are recognized by the patients who will be using the POS in the target language.

Achieving conceptual definition and equivalence forms the foundation for the cross-cultural adaptation and translation process as it helps to clarify conceptual elements that are essential for the best pre-final version to be constructed after phase IV. Hence, keeping detailed records of all three steps is quite important.

Key points:

- Brief review of literature on health related quality of life concepts of target language/culture
- Discussion of key concepts underlying each item of the original POS with palliative care professionals
- Discussion of concepts defined previously with palliative care patients
- Keep records of challenging concepts, uncertainties and rationale of final decisions for each step

Phase II: Forward Translation

Forward translation (FT) is a term to refer to the process of translating the Original POS[1] (or one of its derivatives) into the target language. FT is the step to complete after achieving conceptual definition or equivalence. This step is required before a backward translation can be completed (use Appendices C and D).

Translation of the original POS to the target language involves three translators and two main steps. First, two forward translations to the target language, completed independently by two translators, are required. Two translations allow for comparisons to be made between the translations, and for discrepancies to be identified, e.g. differences in how a word and/or a phrase has been translated. Discussing discrepancies allows for the best choice or words to result, and therefore also the best translation between the two translations.

To aid the process of the forward translation and to create an audit trail of the adaptation and translation process, each translator produces a written report of the translation done independently. A record of the challenging phrases or uncertainties as well as the rationale for final choices needs to be included in this report. Reports are generated for the scale itself and the instructions and the scoring documentation. It is important that both translators have different profiles or backgrounds to ensure the best possible translation and that both medical and usual spoken language with its cultural nuances is present.

The two independent translations should be produced by bilingual translators who have the target language as their mother tongue to accurately reflect the nuances of that language. The translators do not need to be certified but should hold complementary backgrounds.

Box 2

Forward Translator 1: Ideally, one of the translators should be knowledgeable about the type of concepts present in the POS, i.e., health and palliative care terminology and the content area of construct. Translator 1 adaptations will be aimed at equivalency from a more clinical perspective. The Forward Translation 1 (FT1) will be created.

Forward Translator 2: The other translator should, preferably, have no medical/clinical background. As the so-called “naive” translator, he or she is more likely to detect the more subtle differences in meaning to the original and offer a translation that reflects the language used by the common population. The Forward Translation 2 (FT2) will be created.

It is likely that both translators will naturally belong to a medium-high cultural status and some biases/ tendencies toward various types of translations may occur. Hence,

the second step is important for consensus-building as it helps minimize biases/tendencies toward certain types of translations.

To produce one common translation by synthesizing Forward Translation 1 and Forward Translation 2 (FT1_2), a third person is added to the team at this point. This person will serve as a mediator in discussions of translation differences, and will produce a written documentation of this phase. Working from the original questionnaire as well as the first and the second translator's versions a new translation is produced: FT1_2. The written report will document the synthesis process, addressing discussed issues and how these were resolved. It is important that all issues are resolved by consensus from all parties and that the entire process is well documented.

Key Points:

- Translator 1: clinician with knowledge of palliative care, native speaker in target language produces FT1
- Translator 2: no Medical background (naive) native speaker in target language produces FT2
- Translators 1 and 2 work independently
- Synthesising both translations into one with the help from a third person – mediator - to produce FT1_2
- Keep a record, for each FT, of challenging phrases, uncertainties and rationale of final decisions

Phase III: (Blind) Backward translation

Backward translation (BT) is a term that refers to the process of translating the target language version back to English.

Here we describe the optimum process which involves two backward translations (use Appendices C and E); with the BT completed by two independent translators blinded to the original English version. However, it is also acceptable to have a minimum of one backward translation by someone who is blinded to the original POS.

BT involves a process of validity checking which ensures that the new translated version accurately reflects the item content of the original version. However, agreement between the BT and the original source version does not guarantee a satisfactory FT1_2, because a consistent translation does not mean that it is correct. BT works as one type of validity check, which helps to identify gross inconsistencies or conceptual errors in the translation.

Similarly to the process of FT, BT1 and BT2 are produced by two bilingual persons, but this time the source language (English) is their mother tongue. The two translators should be blinded (neither be aware nor be informed) to the original POS and concepts explored, thus avoiding information bias.

Box 3

Backward Translator 1: One of the translators should be knowledgeable about the type of concepts present in the POS, so, health and palliative care terminology and the content area of construct. Translator number 1's adaptations will be aimed at equivalency from a more clinical perspective. The Backward Translation 1 is produced.

Backward Translator 2: The other translator should, preferably, have no medical/clinical background. As the so-called "naive" translator, they are more likely to detect the more subtle differences in meaning of the original and offer a translation that reflects the language used by the common population. The Backward Translation 2 is produced.

Key Points

- Back Translator 1: clinician with knowledge of palliative care, native speaker in english
- Back Translator 2: no Medical background (naive) native speaker in english
- Back Translators 1 and 2 work independently from the FT1_2 to produce one translation each in english
- Synthesising both back translations into one with the help of a third person mediator
- Keep a record of challenging phrases, uncertainties and rationale of final decisions

Phase IV: Expert Review

The aim of the Expert Review committee is to discuss and resolve any ambiguities between a) the BTs to produce a final BT; and b) the final BT and the original POS. This is done through consensus among all committee members to derive a pre-final version of the POS in the target language (use Appendix C).

The composition of the Expert Committee is crucial to achieving cross-cultural equivalence of the translated instrument. To produce one common backward translation (BT 1_2) by synthesizing the two backward translations (BT1 and BT2), a multi-disciplinary committee including a member from the research team, one health care professional familiar with the content areas and construct of the POS and all four translators should be involved in the process. If a face to face meeting isn't possible, a telephone conference can be organized and e-mails should circulate among all so that all comments are taken into account.

Box 4

Using this methodology, this pre-final version will have initial **conceptual, semantic, experiential and content equivalence.**

Conceptual: degree to which a concept of the POS items exists in both cultures and the meaning is the same, i.e., "family" may be thought as a nuclear unit in one culture (parents and offspring only) and extended (other members) in another.

Semantic: sentence structure, colloquialisms or idioms which ensure the meaning of the text or idea of the items.

Experiential: items seeking to capture experience of daily life often vary in different countries and cultures. In some instances, a given task may simply not be experienced in the target culture, even if it is translatable. To address this situation, a questionnaire item addressing a similar action or intent in the target culture would need to be identified to replace the original item.

Content equivalence: relevance or pertinence of the text or idea of the items in each culture.

The Expert Committee's role is to evaluate, revise and consolidate the instructions, items and response format of the backward translated POS to develop the pre-final version ready for psychometric testing.

Corresponding written reports created during previous phases (explaining the rationale of each decision) should be available (even if not all are circulated). Decisions will need to be based on those documents.

It may happen that two different suggestions for a certain item may seem equally appropriate. However, the final decision does not have to happen in this phase. The next phase, cognitive debriefing, will be another aid to help with decision making. Again, keeping a robust record of discussion, rationale and reasons of uncertainties are crucial.

Finally, the back translated English versions may be in native British, American, Australian or any other version of the English language. These differences can be acknowledged, but there is no need to agree upon the best terminology in these cases.

Key points:

- BT1 and BT2 are combined to produce a final BT1_2 (not applicable if there is only one Back Translation)
- Final BT1_2 is compared with original POS and differences need to be discussed and resolved
- Records of previous phases should be available for consultation
- The pre-final POS in the target language is produced
- Keep a record of challenging phrases, uncertainties and rationale of final decisions

Phase V: Cognitive debriefing

Cognitive debriefing sometimes referred to as cognitive interviewing is a term to describe a qualitative pretesting of the new POS in the target language, ensuring that the original instructions, items and scoring materials are clearly expressed. Two interviewers (usually the coordinator and someone to assist) are needed.

This field test of the new questionnaire uses the pre-final version with patients and health care providers. These groups are independent and therefore happen at different times. Each group should ideally have between 5 to 8 participants. Each subject first completes the questionnaire, and is then asked about their thoughts on what was meant by each item and their response. Both the meaning of the items and responses are to be explored. This retrospective approach provides useful data regarding how an individual person interprets the items on the questionnaire, as well as their overall comprehension of the measure. It does not, however, addresses the construct validity, reliability or item response patterns. These will be studied in phase VII. The described process provides for assessment of quality in the content validity.

Ideally, a purposive sample of the target population is interviewed to ensure a wide range of responses regarding the quality of the new version. The views of patients and health care professionals are an indicator of face validity. Ideally, inclusion criteria should be native-speakingpatients living in the target country or culture, fluent in the target language, and currently or previously receiving palliative care. Exclusion criteria should be those with a cognitive or communication impairment or a physical limitation (e.g., exclude because of fatigue and not because of assisted communication needs), unable to participate in data collection and/or an expectation of death within days (if too frail or ill). Professionals to be interviewed should have extensive experience in palliative care and be actively working in different palliative care settings.

Box 5
The semi-structured interview or focus group should address:
1) the interviewees' comprehension of the instructions, each item and its response options; questionnaire clarity, difficulties in understanding and answering the POS questions, length, and overall relevance of the questionnaire for their health problem;
2) specific questions related to the reason why any question was difficult to understand or to be answered;
3) asking the participants for suggestions as to how to rewrite the statements that are identified as unclear or not appropriate.

Appendix B completed in Phase I should be available for consultation if needed. Every item should be appraised by both interviewers for qualitative appraisal of comprehensiveness, acceptability, relevance, comparability, and interpretability. Appreciation of potential culturally inappropriate items for members of the target culture or for items dealing with taboo subjects is encouraged. (This is why item 7 has two versions: version 1 asks about life being worthwhile and version 2 asks about

depression. Both items should be translated and validated so that there is a choice of use, depending on the context in which POS will be used).
Time of completion of the POS should also be recorded as this enables the assessment of the applicability of POS.

Key points:

- Semi structured interviews are conducted with patients
- Semi structured interviews are conducted with palliative care professionals
- Comprehension, interpretability and suggestions of improvement are to be discussed
- Development of the final POS in the target language ready for psychometric testing

PHASE VI: Proof reading

At this point, all completed templates describing each phase of the process, as well as any questions, should be sent to the POS Development Team to proof read and endorse before psychometric testing/validation.

The POS Development Team will look for inconsistencies in the process as well as looking at the format of the new POS translation and will address any questions that might arise.

All documents are to be sent by e-mail to palliativecare@pos-pal.org

Phase VII: Psychometric testing/validation

Before the psychometric testing it is necessary to collect data. It is recommended that you spend some time thinking about practicalities and logistics of data collection. It is also crucial to check if ethics approval and informed consent from patients are needed. Consider what other validated measures already exist in the target language, because you will need to ask subjects to fill the POS along with those measures to estimate construct validity. A very long set of measures is not recommended. However, it is possible that in some target languages no other measures in palliative care exist. In that case, please consider searching in the oncology literature for a measure capturing quality of life and symptoms.

Box 6
It is helpful to think about and consider:

- Will you be doing the validation for patients only or will be doing it for staff and/or family as well?

- What demographics will be collected?

- Will the data be collected in one site only or several centres? There are practical implications that need to be addressed to make sure that the measure is being used in a valid and standardised way across centres. Meetings need to be held with management and actual staff who will be giving the measure to patients. Staff may also need a brief session on what to do and how to explain patients why they are asking them to complete the POS. You also need to think about how many patients you need in each centre.

- Depending on the setting, how often will you have the same patient coming in? This is important to test for reproducibility of the new POS. So there is a need to think about the interval between measurements on the same patient and how you achieve those.

- It might be interesting to record, during data collection, why there was missing data, i.e., patient was too tired to finish, did not understand the question, felt the question was not applicable to their situation, etc. Consider asking staff who will get the questionnaires from the patients to do it.

The aim of psychometric testing is to ensure that the new version has demonstrated the measurement properties needed to obtain reliable and valid results from its application.

The sample size for this step depends on the types of psychometric approaches that will be used. The more complete the psychometric approaches for evaluation of the translated instrument the more confidence will be generated in its reliability and

validity properties. In general, per rule of thumb, it is highly recommended to use a minimum of 10 subjects per item of the instrument scale. An excellent number would be between 150 and 200 subjects, but we recognize that it can be difficult to recruit this number of patients in palliative care.

After checking and cleaning your data you will need to test it for normality which will help you decide what tests to use. Always treat POS data as continuous or ordinal. Please see published studies on POS validation and references provided in this document as well as other literature regarding the most suitable tests to be performed. **It is your responsibility** to choose the correct ones, depending on the type of data you have. Also, consider what approach you will decide on handling missing data.

The most recommended and commonly used psychometric approaches in this step are estimation of:

1. **Internal consistency reliability**: a measure of the extent to which items in a questionnaire are correlated, thus measuring the same construct.

2. **Test-retest reliability** (reproducibility): it concerns the degree to which repeated measurements in stable people provide similar results (or answers). So, only patients attending a palliative care service for a period of time and designated by staff as clinically stable should complete the new POS and the time frame should be close in duration to ensure that no changes have occurred. Therefore, you need to consider the clinical status of your subjects: is your population relatively stable – wouldn't change substantially over time – or is your population expected to change rapidly over time. This will help to decide what (1) what time points you will include in your analysis (consecutive time points or baseline and a time point further ahead) and (2) what is your time frame between assessments (is it going to be 2 days or 1 week?). Patients with clinical changes between the two assessments should be excluded from analysis. This measurement can be calculated by weighted kappa agreement and intra-class correlations.

3. **Construct validity**: the extent to which scores in the new instrument relate to other validated measures already existent in the target language that measure the same concepts. This measurement is assessed by testing predefined theoretical hypothesis about expected correlations or differences. Patients are asked to complete all measures consecutively on a single occasion. In 2010 Siegert et al[9] published a study on a factor-analytic examination and concluded that the 10 POS items appear to cover 5 domains: pain, symptoms, well-being, family anxiety and quality of care. Therefore, the existing measures in the target language that will be given to patients with the POS, need to cover those dimensions.

4. **Criterion validity** (concurrent and/or predictive validity): it refers to the extent to which scores on the new questionnaire relate to a gold standard (a diagnostic test or benchmark that is the best available for a situation in a certain context).

5. **Responsiveness to change:** the ability to detect small clinically important changes over time. It is related to longitudinal validity, in other words, it discriminates between important changes and measurement error over time (i.e. between measurements) no matter how small these changes were. Comparisons between first and the second, and the first and third consecutive evaluations can be undertaken for staff and patients' ratings. Longitudinal behaviour of every response and its confidence intervals (CIs) can be calculated. The standardized effect size and the standardized response mean may also be computed.

6. The answers to the open question in item 11 are to be content analyzed and categorised if possible. (See Hsieh 2005 in other references).

Key points:

- Before data collection consider logistics and practicalities
- Go through literature to decide what statistics you will use depending on type of data and number of subjects. Always treat POS data as continuous or ordinal. Think about how to deal with missing data
- Test the new POS for: internal consistency, reliability, construct validity, criterion validity and responsiveness to change
- Analyse item 11 by performing content analysis and categorisation, if possible.

Phase VIII: Report and publication

The final version of a written report describing the results of the validation and psychometric testing needs to be proof read by the POS Development Team. After that, we strongly encourage authors to write and submit the full study for publication. Consider using the COSMIN check list to aid you in writing your manuscript.

The new POS measure will be uploaded on the POS website to be available to all interested in using it. The publication will be part of the POS publication list on the POS website[10] with the link for the digital format.

References

1. Hearn J, Higginson IJ. Development and validation of a core outcome measure for palliative care: The Palliative Care Outcome Scale. Palliative Care Core Audit Project Advisory Group. Quality in Health Care 1999;8(4):219–227.

2. Beaton D, Bombardier C, Guillermin F, Ferraz MB, Recommendations for the Cross-Cultural Adaptation of Health Status Measures, Supported by the American Academy of Orthopaedic Surgeons, Institute for Work & Health, Revised March 2002

3. Beaton D, Bombardier C, Guillermin F, Ferraz MB, Recommendations for the Cross-Cultural Adaptation of Health Status Measures. Spine 2000, Vol 25 (24), 3186-2191

4. Guillemin F, Bombardier C and Beaton D, Cross-cultural adaptation of health-related quality if life measures: literature review and proposed guidelines, J Clin Epidemiol 1993, vol 46 (12), 1417-1432

5. www.mapi-institute.com

6. Cull A, Sprangers M, Bjordal K, Aaronson N, West K and Bottomley A on behalf of the EORTC Quality of Life Group, EORTC Quality of Life Group Translation Procedure, 2nd Edition, February 2002.

7. Sousa VD, Rojjanasrirat W, Translation, adaptation and validation of instruments or scales for use in cross-cultural health care research: a clear and user-friendly guideline. Journal of Evaluation in Clinical Practice 17 (2011) 268-274

8. Terwee CB, Bot SDM, de Boer MR, et al, Quality criteria were proposed for measurement properties of health status questionnaires, J Clin Epi 2006, vol 60, 34-42.

9. Siegert RJ, Gao W, Walkey FH, Higginson IJ. Psychological well-being and quality of care: a factor-analytic examination of the palliative care outcome scale. Journal of Pain & Symptom Management. 2010;40(1):67-74.

10. pos-pal,org

Additional references:

Bausewein C et al., Validation and Clinical Application of the German Version of the Palliative Care Outcome Scale, J Pain Symptom Manage 2005; 30:51–62.

Bausewein C, Daveson B, Benalia H, Simon ST, Higginson IJ. Outcome Measurement in Palliative Care. PRISMA, London 2011. (http://pos-pal.org/Resources.php)

De Vet HCW, Terwee CB, Mokkink LB, Knol DL. Measurement in Medicine. 1st ed. Cambridge: Cambridge University Press; 2011.

Eisenchlas JH et al, Use of the Palliative Outcome Scale in Argentina: a cross-cultural adaptation and validation study, J Pain and Symptom Manage 2008; 35; 188-202.

Hsieh H-F, Shannon SE. Three approaches to qualitative content analysis. Qual Health Res. 2005;15(9):1277-88

Mokkink LB, Terwee CB, Patrick DL, Alonso J, Stratford PW, Knol DL, et al. The COSMIN checklist for assessing the methodological quality of studies on measurement properties of health status measurement instruments: an international Delphi study. Qual Life Res. 2010;19(4):539-49.

Mokkink LB, Terwee CB, Patrick DL, Alonso J, Stratford PW, Knol DL, et al. The COSMIN study reached international consensus on taxonomy, terminology, and definitions of measurement properties for health-related patient-reported outcomes. J Clin Epidemiol. 2010;63(7):737-45

Please visit the POS website for a complete reference list of POS publications at: pos-pal.org

Acknowledgements:

We thank Natália Calanzani and Hristina Petkova for proof reading and providing comments as users of this manual.

Appendices

These Appendices are available on-line, in word format so that you may change them to better suit your needs (i.e. add or remove rows from tables)

Please visit pos-pal.org

Report on the Cross-Cultural Adaptation and Validation of the Palliative care Outcome Scale (POS)

Brief Questionnaire (for POS Development Team records)

1. What scientific group do you belong to and where is it based (i.e. University, etc)?
2. Are you involved in Palliative Care? How?
3. What is your nationality?
4. Do you have a research or clinical background (or both)?
5. What is your research experience?
6. Have you got any previous experience in validating a Patient reported outcome measure?
7. Are you a student and would this validation be part of your studies (i.e. MSc project)? Who is your supervisor?
8. Where and how will you use the translated and validated POS?
9. Do you have access to patients for the validation process?

Submission Date: _________________ (dd/mm//yyyy)

POS Developing Team Acceptance Date: ____________________ (dd/mm/yyyy)

Applicant Name:
____

Applicant Institution:
____

Applicant Address:
____
Street, Post code
____
City, Country
____

Phone: +_________________ Fax: +______________________

Email:_________________________________

POS Questionnaire for validation: POS ☐ POS-S ☐ POS + POS-S ☐

Target Group/Population Information:

Country: ______________________ Culture: _________________

Language: ____________________

Patient Population: ______________________

Translation Participants

Phase	Name	Qualifications/Title
FT1 Translator		
FT2 Translator		
T1&2 Mediator		
BT1 Translator		
BT2 Translator		
**Other Members of Expert Committee (in addition to translators)**		
Methodologist		
Clinician		
Language Expert		
Service Users		
Other:		
Pre-Test Coordinator		
Psychometric Testing Coordinator		

Documentation

Appendix A
Summary of all phases of the process

Phase	Documents	Date of completion
I: Conceptual Definition	Appendix B	
II: Forward Translation (FT)	Appendices C and D	
	FT1: Translation to target language	
	FT1: Keep a record	
	FT2: Translation to target language	
	FT2: Keep a record	
	FT1&FT2 agreed version	
	Final Report – contact POS Team	
III: Backward Translation (BT) (blinded to the FT and the original POS)	Appendices C and E	
	BT1 Translation from target language to English	
	BT1 Keep a record	
	BT2 Translation from target language to English	
	BT2 Keep a record	
IV: Expert review	Appendix C	
	Pre-Final translation	
	Final Report – contact POS Team	
V: Conceptual Cognitive Debriefing (sometimes referred to as pre-testing or cognitive interviewing)	Appendices C and the new POS in the target language (to be created)	
	Ethics Approval (depends on your working context, make sure you ask)	
	Cognitive Interviews with patients and health care professionals (minimum 5 for each group) Multiple sites and multiple conditions may make a more robust study, but you will also need more time and effort	
	Final Report – contact POS Team	
VI: Proof Reading by POS Development Team	All completed Appendices to be sent by email	
VII: Psychometric testing (n=)	Appendix F	
	1. Internal consistency reliability	
	2. Test-retest reliability (reproducibility)	
	3. Construct validity	
	4. Criterion validity	
	5. Responsiveness to change	
	Final Report – contact POS Team	
VIII: Report and publication		

Appendix B
Phase I – Conceptual Definition

Original questionnaire:	Conceptual Definitions/meaning
Please answer the following questions by ticking the box next to the answer that is most true for you. Your answers will help us to keep improving your care and the care of others. Thank you.	
**1. Over the past 3 days, have you been affected by pain?**<br>0 Not at all, no effect<br>1 Slightly - but not bothered to be rid of it<br>2 Moderately - pain limits some activity<br>3 Severely - activities or concentration markedly affected<br>4 Overwhelmingly - unable to think of anything else	
**2. Over the past 3 days, have other symptoms e.g. nausea, coughing or constipation seemed to be affecting how you feel?**<br>0 No, not at all<br>1 Slightly<br>2 Moderately<br>3 Severely<br>4 Overwhelmingly	
**3. Over the past 3 days, have you been feeling anxious or worried about your illness or treatment?**<br>0 No, not at all<br>1 Occasionally<br>2 Sometimes - affects my concentration now and then<br>3 Most of the time - often affects my concentration<br>4 Can’t think of anything else - completely pre-occupied by worry and anxiety	
**4. Over the past 3 days, have any of your family or friends been anxious or worried about you?**<br>0 No, not at all<br>1 Occasionally<br>2 Sometimes – it seems to affect their concentration<br>3 Most of the time	

4 Yes, always preoccupied with worry about me	
**5. Over the past 3 days, how much information have you and your family or friends been given?**<br>0 Full information or as much as wanted – always feel free to ask<br>1 Information given but hard to understand<br>2 Information given on request but would have liked more<br>3 Very little given and some questions were avoided<br>4 None at all – when we wanted information	
**6. Over the past 3 days, have you been able to share how you are feeling with your family or friends?**<br>0 Yes, as much as I wanted to<br>1 Most of the time<br>2 Sometimes<br>3 Occasionally<br>4 No, not at all with anyone	
**7. Version 1 - Over the past 3 days, have you felt that life was worthwhile?**<br>0 Yes, all the time<br>1 Most of the time<br>2 Sometimes<br>3 Occasionally<br>4 No, not at all	
**7. Version 2 - Over the past 3 days, have you been feeling depressed?**<br>0 No, not at all<br>1 Occasionally<br>2 Sometimes<br>3 Most of the time<br>4 Yes, all the time	
**8. Over the past 3 days, have you felt good about yourself as person?**<br>0 Yes, all the time<br>1 Most of the time<br>2 Sometimes<br>3 Occasionally<br>4 No, not at all	
**9. Over the past 3 days, how much time do you feel has been wasted on appointments relating to your**	

**healthcare, e.g. waiting around for transport or repeating tests?**<br>0 None at all<br>2 Up to half a day wasted<br>4 More than half a day wasted	
**10. Over the past 3 days, have any practical matters resulting from your illness, either financial or personal, been addressed?**<br>0 Practical problems have been addressed and my affairs are as up to date as I would wish<br>2 Practical problems are in the process of being addressed<br>4 Practical problems exist which were not addressed<br>0 I have had had no practical problems	
**11. If any, what have been your main problems in the last 3 days?**<br>1.<br>2.	
**12. How did you complete this questionnaire?**<br>0 On my own<br>1 With the help of a friend or relative<br>2 With the help from a member of staff	

Appendix C
Final Report for Phases II to V – agreed version(s)

Phase:	
Date, Place:	
People involved:	
Items that did not required discussion	
Discrepancies and their resolution	
Issue: specify item and describe issue	Resolution

Appendix D

Forward Translation: to be given to both independent translators

This template should be used for all respondents' questionnaires – patient, family and caregiver - once the process is finalised, only the subject and verb have to be changed in order to accommodate different respondents)

You may change the information asked to the patient in the header, according to your setting needs, please look at the original English version.

Original English version	New Target Language:
Please answer the following questions by ticking the box next to the answer that is most true for you. Your answers will help us to keep improving your care and the care of others. Thank you.	
**1. Over the past 3 days, have you been affected by pain?**<br>0 Not at all, no effect<br>1 Slightly - but not bothered to be rid of it<br>2 Moderately - pain limits some activity<br>3 Severely - activities or concentration markedly affected<br>4 Overwhelmingly - unable to think of anything else	
**2. Over the past 3 days, have other symptoms e.g. nausea, coughing or constipation seemed to be affecting how you feel?**<br>0 No, not at all<br>1 Slightly<br>2 Moderately<br>3 Severely<br>4 Overwhelmingly	
**3. Over the past 3 days, have you been feeling anxious or worried about your illness or treatment?**<br>0 No, not at all<br>1 Occasionally<br>2 Sometimes - affects my concentration now and then<br>3 Most of the time - often affects my concentration<br>4 Can't think of anything else - completely pre-occupied by worry and anxiety	
**4. Over the past 3 days, have any of your**	

**family or friends been anxious or worried about you?**<br>0 No, not at all<br>1 Occasionally<br>2 Sometimes – it seems to affect their concentration<br>3 Most of the time<br>4 Yes, always preoccupied with worry about me	
**5. Over the past 3 days, how much information have you and your family or friends been given?**<br>0 Full information or as much as wanted – always feel free to ask<br>1 Information given but hard to understand<br>2 Information given on request but would have liked more<br>3 Very little given and some questions were avoided<br>4 None at all – when we wanted information	
**6. Over the past 3 days, have you been able to share how you are feeling with your family or friends?**<br>0 Yes, as much as I wanted to<br>1 Most of the time<br>2 Sometimes<br>3 Occasionally<br>4 No, not at all with anyone	
**7. Version 1 - Over the past 3 days, have you felt that life was worthwhile?**<br>0 Yes, all the time<br>1 Most of the time<br>2 Sometimes<br>3 Occasionally<br>4 No, not at all	
**7. Version 2 - Over the past 3 days, have you been feeling depressed?**<br>0 No, not at all<br>1 Occasionally<br>2 Sometimes<br>3 Most of the time<br>4 Yes, all the time	
**8. Over the past 3 days, have you felt good about yourself as person?**<br>0 Yes, all the time	

1 Most of the time<br>2 Sometimes<br>3 Occasionally<br>4 No, not at all	
**9. Over the past 3 days, how much time do you feel has been wasted on appointments relating to your healthcare, e.g. waiting around for transport or repeating tests?**<br>0 None at all<br>2 Up to half a day wasted<br>4 More than half a day wasted	
**10. Over the past 3 days, have any practical matters resulting from your illness, either financial or personal, been addressed?**<br>0 Practical problems have been addressed and my affairs are as up to date as I would wish<br>2 Practical problems are in the process of being addressed<br>4 Practical problems exist which were not addressed<br>0 I have had had no practical problems	
**11. If any, what have been your main problems in the last 3 days?**<br>1.<br>2.	
**12. How did you complete this questionnaire?**<br>0 On my own<br>1 With the help of a friend or relative<br>2 With the help from a member of staff	

Appendix E

Backward Translation: **DO NOT** provide this template to the independent translators. They should be blinded to the original POS and the forward translations.

(This template should be used for all respondents' questionnaires – patient, family and caregiver - once the process is finalised, only the subject and verb have to be changed in order to accommodate different respondents)

The Backward translation is done ***without looking*** at this template or the original POS. Once the BTs are done, you may copy them to the blank column so that it is easier to compare results.

Original English POS	Backward Translation from target language
Please answer the following questions by ticking the box next to the answer that is most true for you. Your answers will help us to keep improving your care and the care of others. Thank you.	
**1. Over the past 3 days, have you been affected by pain?**<br>0 Not at all, no effect<br>1 Slightly - but not bothered to be rid of it<br>2 Moderately - pain limits some activity<br>3 Severely - activities or concentration markedly affected<br>4 Overwhelmingly - unable to think of anything else	
**2. Over the past 3 days, have other symptoms e.g. nausea, coughing or constipation seemed to be affecting how you feel?**<br>0 No, not at all<br>1 Slightly<br>2 Moderately<br>3 Severely<br>4 Overwhelmingly	
**3. Over the past 3 days, have you been feeling anxious or worried about your illness or treatment?**<br>0 No, not at all<br>1 Occasionally<br>2 Sometimes - affects my concentration now and then<br>3 Most of the time - often affects my concentration	

4 Can't think of anything else - completely pre-occupied by worry and anxiety	
**4. Over the past 3 days, have any of your family or friends been anxious or worried about you?**<br>0 No, not at all<br>1 Occasionally<br>2 Sometimes – it seems to affect their concentration<br>3 Most of the time<br>4 Yes, always preoccupied with worry about me	
**5. Over the past 3 days, how much information have you and your family or friends been given?**<br>0 Full information or as much as wanted – always feel free to ask<br>1 Information given but hard to understand<br>2 Information given on request but would have liked more<br>3 Very little given and some questions were avoided<br>4 None at all – when we wanted information	
**6. Over the past 3 days, have you been able to share how you are feeling with your family or friends?**<br>0 Yes, as much as I wanted to<br>1 Most of the time<br>2 Sometimes<br>3 Occasionally<br>4 No, not at all with anyone	
**7. Version 1 - Over the past 3 days, have you felt that life was worthwhile?**<br>0 Yes, all the time<br>1 Most of the time<br>2 Sometimes<br>3 Occasionally<br>4 No, not at all	
**7. Version 2 - Over the past 3 days, have you been feeling depressed?**<br>0 No, not at all<br>1 Occasionally<br>2 Sometimes<br>3 Most of the time<br>4 Yes, all the time	

**8. Over the past 3 days, have you felt good about yourself as person?**<br>0 Yes, all the time<br>1 Most of the time<br>2 Sometimes<br>3 Occasionally<br>4 No, not at all	
**9. Over the past 3 days, how much time do you feel has been wasted on appointments relating to your healthcare, e.g. waiting around for transport or repeating tests?**<br>0 None at all<br>2 Up to half a day wasted<br>4 More than half a day wasted	
**10. Over the past 3 days, have any practical matters resulting from your illness, either financial or personal, been addressed?**<br>0 Practical problems have been addressed and my affairs are as up to date as I would wish<br>2 Practical problems are in the process of being addressed<br>4 Practical problems exist which were not addressed<br>0 I have had had no practical problems	
**11. If any, what have been your main problems in the last 3 days?**<br>1.<br>2.	
**12. How did you complete this questionnaire?**<br>0 On my own<br>1 With the help of a friend or relative<br>2 With the help from a member of staff	

Appendix F

Psychometric testing report.

Sample description	
Sample Size:	
Patients' condition(s)	
Age: (mean, std deviation)	
Gender (M, F)	
Study description	
**Internal consistency**<br>Please describe methods used and results	
**Test-retest Reliability**<br>Please describe methods used and results	
**Construct Validity**<br>Please describe methods used and results	
**Criterion validity**<br>Please describe methods used and results	
**Responsiveness**<br>Please describe methods used and results	
**Other psychometric testing**<br>Please describe methods and results	
**Please describe overall results:**	
