# Supplementary material for: Translation and cognitive testing of the Italian Integrated Palliative Outcome Scale (IPOS) among patients and healthcare professionals
Source: PLoS One. 2019 Jan 2;14(1):e0208536. doi: 10.1371/journal.pone.0208536 (PMC6314603; doi:10.1371/journal.pone.0208536)
Supplement: S3 Appendix — (DOCX) [file pone.0208536.s003.docx]

| ISSUES | ORIGINAL VERSION | VERSION ADOPTED IN THE IPOS_ITA COGNITIVE PHASE | COMMENTS RAISED UP IN THE COGNITIVE INTERVIEWS-FOCUS GROUPS | COMMENTS RAISED UP IN THE AUDIT | FINAL VERSION CHANGES AND REFORMULATIONS |
| --- | --- | --- | --- | --- | --- |
| Date field  In the original IPOS English the date field indicates the date of compilation of the questionnaire.  Some patients and professionals filled it with the date of birth | DATE | DATA (DATE) | Confusion between date of birth and the date of the questionnaire completion | Participants agreed that was acceptable to put DATA ODIERNA (Today’s date) | DATA ODIERNA |
| Layout issues  In different sections of the IPOS some layout issues were raised |  |  | Some patients complained for too small line-spacing for open questions, small characters and confusion due to the lack of distance between different sections causing misleading in the answers (the order of possible answers changes between Q5 and Q6) |  | The line spacing for the open questions and graphic characters were augmented.  A spacing between Q5 and Q6 was added, now the groups of questions are clearly parted and related to the correct answers |
| Q2.  The question  The possible answers about how much the symptoms affected the patients overtime, did not allow participants to report clearly in case of fluctuations | Below is a list of symptoms, which you may or may not have experienced. For each  symptom, please tick one box that best describes how it has affected you over the past  week. |  | Patients and professional were uncertain when had to decide what intensity to choose in case of fluctuating symptoms in the investigated time. A specification sentence was required to better describe the experience of the symptom. | The audit group confirmed that this specification could be of help. Participants agreed that this change should be considered for other IPOS and POS versions | Q2. (…) NEL CASO IL SINTOMO ABBIA AVUTO DELLE FLUTTUAZIONI INDICARE UN VALORE MEDIO  (if the symptom had a fluctuating course, indicate a mean value) |
| Q2.  The answers  Lack of concordance between IPOS_ITA and the existing POS_ITA versions | - Not at all - Slightly - Moderately - Severely - Overwhelmingly | According to the author’s requests the following possible answers were chosen:   - NO, PER NULLA - LIEVEMENTE - MODERATAMENTE - IN MODO SEVERO - IN MODO OPPRIMENTE |  | The difference between “No per nulla” and “no, per niente” was discussed. Probably in Italian there is no difference in meaning. Having the POS_ITA[[1](#_ENREF_1)] been fully validated the group decides to keep the POS_ITA wording, therefore this will change in IPOS_ITA  For the same reasons “lievemente” will be reworded with “leggermente” and “opprimente” with “intollerabile” | - NO, PER NIENTE - LEGGERMENTE - MODERATAMENTE - IN MODO SEVERO - IN MODO INTOLLERABILE |
|  |  |  |  |  |  |
| Q2. Question about oral symptoms  Two different symptoms to choose in one question | Sore or dry mouth | SENSAZIONE DI BOCCA ASCIUTTA, DOLORE ALLA BOCCA | Some participants were confused by the request to choose between dry mouth and pain in  your mouth. The two symptoms could coexist or not, and with possible different intensity, therefore patients did not know what to answer | Authors agree that this double question generates confusion. Furthermore the recently published [[2](#_ENREF_2)] neurological English version of IPOS adopted “Mouth Problems” .  A new simplified option was chosen | PROBLEMI AL CAVO ORALE |
| Q3. Issue about the wording  Multiple choice in the question:  Feeling  Anxious OR Worried | Have you been feeling anxious or  worried about your illness or  treatment? | SI È SENTITO PREOCCUPATO O ANSIOSO A CAUSA DELLA MALATTIA O DELLE TERAPIE? | Anxiety is seen as a disease, participants and professional prefer “worried”.  The double choice “illness or treatment” seems confusing and ambiguous.  Re-wording:  SI È SENTITO PREOCCUPATO PER LA SUA SALUTE?  \  Did you feel worried for your health? | Although having the advantage of being one single question, does not address the complex issues felt important by the IPOS developing group. For this reason the POS_ITA wording  “SI È SENTITO IN ANSIA O PREOCCUPATO A CAUSA DELLA SUA MALATTIA O DELLE TERAPIE?”  is adopted. | SI È SENTITO IN ANSIA O PREOCCUPATO PER LA SUA MALATTIA O PER LE TERAPIE? |
| Q4. Issue about the wording  Multiple choices in the question:  Anxious OR worried  AND  Family OR friends | Have any of your family or friends  been anxious or worried about  you?   \|  \| \| --- \| | QUALCHE SUO FAMILIARE O AMICO È STATO PREOCCUPATO O IN ANSIA PER LEI? | For the double choice between the symptoms see above.  Many comments from participants on difficulties to choose between family and friends.  The word “CARI” (“dear ones”) was suggested  The new simplified question was:  I SUOI CARI SONO STATI PREOCCUPATI PER LEI? | This new wording should simplify the question because the word “CARI” (dear or loved ones or close ones?) in Italian seems to be clear and acceptable. This addresses the difficulties of various participants who felt confused from the double choice “family or friends”.  For the reasons explained above anxious or worried was mantained | QUALCUNO DEI SUOI CARI È STATO PREOCCUPATO O IN ANSIA PER LEI? |
| Q5.  Issue: meaning of the word depression. | Have you been feeling  depressed? | SI È SENTITO DEPRESSO? | Professionals felt this as a difficult question. Depression can be seen as a diagnosis rather than a symptom.  Furthermore depression was felt by some patients as a stigma.  Suggested the word “sad” or “low mood” as alternatives  SI È SENTITO TRISTE O GIÙ DI MORALE?  did you feel sad or with low mood? | Even though the evidences of the study did not suggest to use the word depression, participant of the audit decide to keep the original formulation for the following reasons:  This question showed a positive role in the screening of depression allowing clinical decisions based on different answers [[3](#_ENREF_3)].  The same question is validated in POS_ITA. | SI È SENTITO DEPRESSO? |
| Q6.  Issue of feeling at peace.  Is the wording confusing? | Have you felt at peace? | SI È SENTITO IN PACE? | The Italian wording of “feeling at peace” was felt confusing by some patients:  *-“Peace? We are not at war!”-*  *-“Peace? Only the dead are at peace!”-*  Participants suggested to add “serene” to clarify the meaning of the question | The new version: “Have you felt serene, at peace with yourself?” was discussed.  According to the POS_ITA it was decided to adopt “Si è sentito in pace con se stesso?”. The word “sereno” is rejected in order to maintain a congruence with the POS:“Have you felt at peace with yourself”. | SI E’ SENTITO IN PACE CON SÈ STESSO? |
| Q7.  Issue about the wording  Multiple choice in the question:  family OR friends  AND  Discussion about the word “Feeling” | Have you been able to share how  you are feeling with your family or  friends as much as you wanted? | HA POTUTO CONDIVIDERE I SUOI SENTIMENTI CON FAMILIARI O AMICI, NEL MODO CHE DESIDERAVA? | The word “CARI” was suggested as a substitute for family and friends as in -Q4-  The Italian sentence “STATI D’ANIMO” (moods, states of mind) seems better than “SENTIMENTI” (feelings) because the former can change rapidly, while the latter seems less sensitive to change) | The word “CARI” was accepted being consistent with the thoughts in Q4.  STATI D’ANIMO was felt more accurate for its sensitivity to change overtime | HA POTUTO CONDIVIDERE I SUOI STATI D’ANIMO CON I SUOI CARI NEL MODO CHE DESIDERAVA? |
| Q8.  Issue about the wording  “Wanted” vs“desired”  “Only disease related?” | Have you had as much  information as you wanted? | HA RICEVUTO TUTTE LE INFORMAZIONI CHE DESIDERAVA? | No issues about the wording:  The concept of “desired information” was felt clear and culturally appropriated  An issue raised by some patients and professionals, was about the kind of desired information. Some asked if they had to be health or disease related or information in general | The word “DESIDERAVA” is accepted being also consistent with Q7 formulation.  After a thorough discussion, it was decided not to add a further specification about the topic of the information, in order not to limit the possible answers.  The chosen version is also consistent with POS_ITA | HA RICEVUTO TUTTE LE INFORMAZIONI CHE DESIDERAVA? |
| Q9: Issue about the wording of the question  “Personal or financial” | Have any practical problems  resulting from your illness  been addressed? (such as  financial or personal) | SONO STATI AFFRONTATI EVENTUALI PROBLEMI PRATICI DERIVANTI DALLA MALATTIA? (PER ESEMPIO FINANZIARI O PERSONALI) | The wording “practical problems” was felt clear. Some confusion was generated by the word "financial” because in Italian this seems strongly associated to the financial and investment area. “Economical problems” were suggested by participants as an explanation to complete the question.  In order to explore social aspects related to the illness, a different term was proposed since the word “personal” was felt confusing.  “social” was elicited by the researchers | The concept of economical problems more acceptable and understandable than financial was felt appropriate and adopted.  Despite the evidence of difficulties in understanding of “personal problems”, the authors preferred to keep this wording rather than introducing the new concept of social problems, since the latter are not reported in the original IPOS nor in POS_ITA | SONO STATI AFFRONTATI EVENTUALI PROBLEMI PRATICI, PERSONALI O ECONOMICI DERIVANTI DALLA MALATTIA? |

Appendix Table. Detailed issues and changes made from the different phases of the IPOS_ITA translation and cognitive debriefing
